# Acute Effects of a Polyphenol-Rich Leaf Extract of *Mangifera indica* L. (Zynamite) on Cognitive Function in Healthy Adults: A Double-Blind, Placebo-Controlled Crossover Study

**DOI:** 10.3390/nu12082194

**Published:** 2020-07-23

**Authors:** Emma L. Wightman, Philippa A. Jackson, Joanne Forster, Julie Khan, Julia C. Wiebe, Nigel Gericke, David O. Kennedy

**Affiliations:** 1 NUTRAN, Northumbria University, Newcastle-upon-Tyne NE1 8ST, UK; emma.l.wightman@northumbria.ac.uk; 2 Brain, Performance and Nutrition Research Centre, Northumbria University, Newcastle-upon-Tyne NE1 8ST, UK; philippa.jackson@northumbria.ac.uk (P.A.J.); jo.forster@northumbria.ac.uk (J.F.); julie.khan@northumbria.ac.uk (J.K.); 3 Nektium Pharma, Agüimes, 35118 Las Palmas de Gran Canaria, Spain; jwiebe@nektium.com (J.C.W.); ngericke@nektium.com (N.G.); 4 Department of Botany and Plant Biotechnology, University of Johannesburg, Auckland Park 2006, Johannesburg 2092, South Africa

**Keywords:** cognition, attention, memory, brain, polyphenols, mangiferin, mango leaf extract

## Abstract

Extracts made from the leaves of the mango food plant (*Mangifera indica* L., *Anacardiaceae*) have a long history of medicinal usage, most likely due to particularly high levels of the polyphenol mangiferin. In rodent models, oral mangiferin protects cognitive function and brain tissue from a number of challenges and modulates cerebro-electrical activity. Recent evidence has confirmed the latter effect in healthy humans following a mangiferin-rich mango leaf extract using quantitative electroencephalography (EEG). The current study therefore investigated the effects of a single dose of mango leaf extract, standardised to contain >60% mangiferin (Zynamite^®^), on cognitive function and mood. This study adopted a double-blind, placebo-controlled cross-over design in which 70 healthy young adults (18 to 45 years) received 300 mg mango leaf extract and a matched placebo, on separate occasions, separated by at least 7 days. On each occasion, cognitive/mood assessments were undertaken pre-dose and at 30 min, 3 h and 5 h post-dose using the Computerised Mental Performance Assessment System (COMPASS) assessment battery and the Profile of Mood States (POMS). The results showed that a single dose of 300 mg mango leaf extract significantly improved performance accuracy across the tasks in the battery, with domain-specific effects seen in terms of enhanced performance on an ‘Accuracy of Attention’ factor and an ‘Episodic Memory’ factor. Performance was also improved across all three tasks (Rapid Visual Information Processing, Serial 3s and Serial 7s subtraction tasks) that make up the Cognitive Demand Battery sub-section of the assessment. All of these cognitive benefits were seen across the post-dose assessments (30 min, 3 h, 5 h). There were no interpretable treatment related effects on mood. These results provide the first demonstration of cognition enhancement following consumption of mango leaf extract and add to previous research showing that polyphenols and polyphenol rich extracts can improve brain function.

## 1. Introduction

The roots, leaves, fruit and bark of the food plant *Mangifera indica* (mango) have a long history of therapeutic use within traditional medicinal systems for a wide range of conditions. For example, extracts, teas and infusions made from mango leaves have been used for the treatment of diabetes, malaria, diseases of the digestive system, lungs, and kidneys, and as a topical treatment for wounds and burns [1]. The bioactivity of mango leaf extracts may be due to particularly high levels [2] of xanthones. This group of polyphenols are found in a restricted group of plant species [3], including members of the *Hypericum* genus that provide us with a number of medicinal herbal extracts [4], but they are rarely consumed in the diet, with only a few exceptions other than mango itself (e.g., [5]). The predominant member of this structural group in mango leaf is mangiferin, a xanthone glucoside that has been shown to have potential anti-inflammatory, antioxidant, immunomodulatory, neuroprotective, antiproliferative, antidiabetic, DNA protective, and hypoglycaemic properties [6,7,8,9,10].

Whilst structurally distinct from the flavonoids and other polyphenols that are ubiquitous in plant derived foods, mangiferin [8,11,12,13,14] likely owes its beneficial bioactivity to some similar mechanisms of action as found in the wider polyphenol group class [15], including interactions with, and modulation of, diverse components of a wide range of mammalian cellular signal transduction pathways. These pathways, in turn, control gene transcription and a plethora of cellular responses, including cell proliferation, apoptosis, and the synthesis of growth factors, and vasodilatory and inflammatory molecules. In the central nervous system, specific additional interactions attributed to polyphenols include direct neurotransmitter and neurotrophin receptor and signalling pathway interactions, and increased synthesis of neurotrophins and vasodilatory molecules, which, in turn, foster angiogenesis/neurogenesis [15,16,17,18,19,20]. These mechanisms potentially underlie the observation of consistent beneficial cardiovascular effects from meta-analyses of multiple intervention studies [21,22,23], and demonstrations of improved cognitive function [24,25,26,27,28], following diverse polyphenols.

In line with these mechanistic cellular effects, rodent studies have demonstrated that a single administration of mangiferin can improve memory in uncompromised rats [29] and that either single doses or extended supplementation with mangiferin can attenuate the memory deficits or depressive/anxiety behaviours associated with a range of brain insults and challenges. This includes the cholinergic antagonist scopolamine [30], sleep deprivation [31], the injection of lipopolysaccharides [32] and aluminium chloride-induced neurotoxicity in mice [9]. Consistent ex vivo evidence focussing on the hippocampus also shows that mangiferin can protect rodent neuronal tissue from the increase in inflammatory cytokines [9,30,31,32] and the decrease in neurotrophins such as brain-derived neurotrophic factor (BDNF) [9,31], associated with multifarious brain insults. Similarly, mangiferin has been shown to protect the rodent brain from lead-induced structural damage and decrease oxidative stress via interactions within the Nrf2 signalling pathways in rats [10].

A number of recent studies have assessed the potential efficacy of a mango leaf extract standardized to a minimum of 60% mangiferin (Zynamite^®^). In terms of physical performance, several of these studies have assessed the ergogenic effects in humans of both acute [33,34,35,36] and longer-term supplementation [34] with this mango leaf extract combined with the polyphenols luteolin or quercetin. This research has demonstrated an improved performance during high intensity exercise [33,34,35], increased brain oxygenation [33,34], maximal aerobic capacity [33], increased muscle oxygen extraction [34,35] and the attenuation of muscle damage and improvements in the time course of decreased muscle performance [37].

With regard to brain function, in rats, oral administration of mango leaf extract attenuated electroencephalography (EEG) power measured via implanted electrodes (frontal cortex, hippocampus, striatum, reticular formation) across the spectra and brain regions under investigation, with the most striking findings in the alpha and beta wavebands. These effects were synergistically increased by the co-administration of caffeine. A concomitant ex vivo study also demonstrated that 7 days supplementation with the mango leaf extract lead to increased hippocampal pyramidal cell excitability [38]. In a subsequent multi-disciplinary series of studies [39], both the ex vivo hippocampal excitability and the attenuation of EEG spectral power across brain regions in rats were confirmed both for mango leaf extract and mangiferin, confirming this polyphenol as the likely active component of the extract. In two subsequent pilot studies (also reported in [39]), both involving 16 healthy young humans, quantitative EEG was employed at rest and during cognitive task performance 90- and 60-min post-dose respectively. In the first study, in comparison to control, mango leaf extract resulted in modest reductions in ‘eyes open’ power in delta and theta power, and a more pronounced increase in power during cognitive task performance, with significant increases in all wavebands across scalp electrodes interrogating the association cortex. These results were supported by more modest EEG changes in the second study, but no evidence of a synergistic relationship with caffeine. Cognitive task performance and mood were not significantly modulated by mango leaf extract.

The extant literature demonstrating functional benefits following polyphenol consumption, and the previous rodent and pilot human studies assessing the effects of mangiferin and mango leaf extract described above, suggest that a mango leaf extract with high levels of the polyphenol mangiferin may exert beneficial effects on human brain function, including the enhancement of cognitive function. The current exploratory, double-blind, placebo-controlled, balanced crossover study therefore assessed the effects of a single dose of mango leaf extract (Zynamite^®^) on cognitive function and psychological state 30 min, 3 h and 5 h post-dose in a large sample of healthy adults.

## 2. Methods

### 2.1. Design

This study adopted a randomised, double-blind, placebo-controlled, balanced crossover design, in which the acute effects of a single dose of 300 mg mango leaf extract and placebo were assessed on cognitive function and psychological state/mood at 30 min, 3 h and 5 h post-dose. All study procedures were reviewed and approved by Northumbria University’s Department of Psychology Ethics Committee (Ref: 17741) and were conducted according to the principles of the Declaration of Helsinki. The trial was pre-registered at ClinicalTrials.gov (NCT04299217).

### 2.2. Participants

The required sample size for the study (N = 72) was calculated (GPower 3.0) on the basis of delivering adequate power (0.8) to detect a small effect size (f = 0.1). The power to detect the anticipated medium effect size (f = 0.25) exceeded 0.95.

A total of 75 participants were randomised. Three participants subsequently withdrew from the study after completing one testing visit. Two participants were removed from the dataset during blind data review due to a persistent inability to achieve performance criteria across tasks.

**Figure 1:**
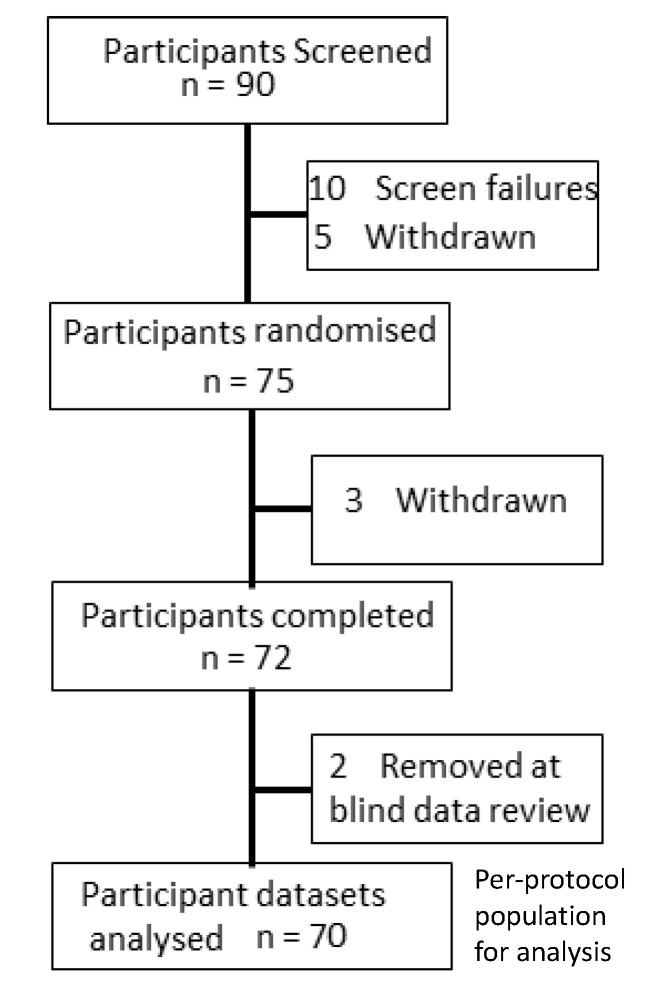
Participant disposition.

The final per-protocol analysis sample therefore comprised 70 participants (F 37/M 33; mean age 26.9 years, range 18–45 years; 5 vegetarians and 1 vegan). All participants self-reported that they were healthy and free from any relevant medical condition or disease, including psychiatric and neurodevelopmental disorders; that they were not taking any prescription or illicit drugs, food supplements or nicotine containing products; that they were not pregnant, lactating or seeking to become pregnant. Participants were also excluded if they consumed >500 mg caffeine per day (>6 × 150 mL cups of filter coffee), had high blood pressure (>systolic 159 mm Hg or diastolic 99 mm Hg) or had a body mass index outside of the range 18.5–35 kg/m^2^. Participant dispositions are shown in Figure 1.

The final number of participants’ data points (excluding missing data and data points removed during blind data review) included in the analysis of data from each individual outcome are shown in the relevant tables.

### 2.3. Treatments

Zynamite^®^ mango leaf extract is comprised of components within the following ranges: mangiferin—60–65%; homomangiferin—3–5%; isomangiferin—up to 1%; leaf polysaccharides—6–20%; hydrolysable and non-hydrolysable tannins—up to 1%; fibre, minerals, moisture—6 to 15%. Details of the manufacturing process are provided elsewhere [39].

Participants were randomly allocated to receive 300 mg mango leaf extract or placebo (maltodextrin) in methylcellulose capsules of identical appearance, during each of their two assessment days. Testing days were separated by a minimum of 7 days to ensure washout. The order in which participants received the two interventions was counterbalanced across the group via random allocation to a counterbalancing schedule. Individual treatments were delivered to the trial facility in individual sealed plastic envelopes, labelled with the participants’ randomisation numbers and visit (1 or 2) according to the computer-generated double-blind randomisation schedule.

There were no significant adverse events that could be linked to administration of the treatments and no significant difference in the incidence of minor adverse events (e.g., mild headache) between the placebo and mango leaf extract treatments.

### 2.4. Psychological Measures

#### 2.4.1. Cognitive Tasks

**Figure 2:**
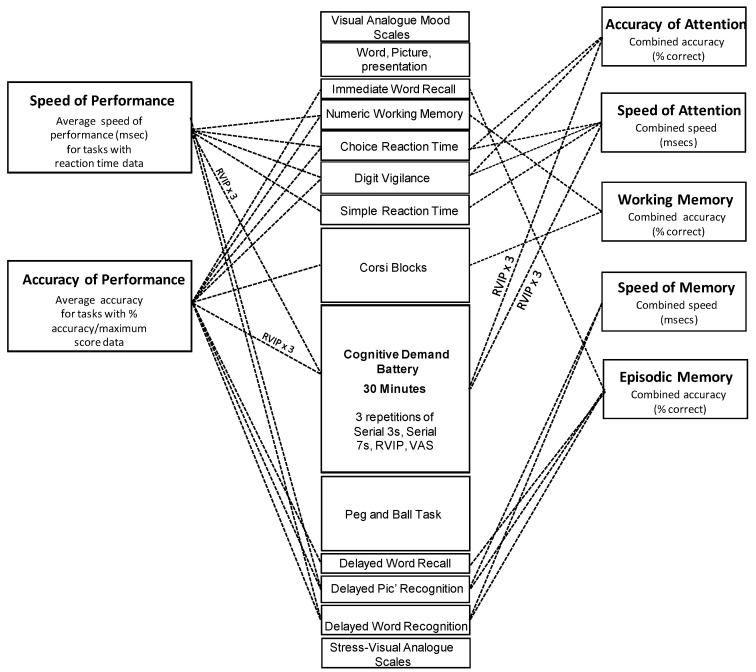
Cognitive assessments. The running order of tasks and their contribution to the cognitive factors (to the right) and global performance measures (to the left) derived from the overall battery. The same assessment was completed at the pre-treatment baseline and at 30 min, 3 h and 5 h post-dose on each assessment day. The selection of tasks took a total of 60 min to complete, with the Cognitive Demand Battery comprising 30 min of this. The individual tasks are described in more detail in the supplementary online materials (Section I). Rapid Visual Information Processing task (RVIP). Visual analogue scale (VAS).

All of computerised cognitive/mood assessments were identical, and were carried out via laptop computers and response boxes using the Computerised Mental Performance Assessment System (COMPASS, Northumbria University, Newcastle upon Tyne, UK). This software platform incorporates the presentation of classic and custom computerised cognitive tasks, with fully randomised parallel versions of each task delivered at each assessment for each individual. A similar selection of tasks has previously been shown to be sensitive to diverse nutritional interventions [40,41,42,43]. Within the 60-min assessment the participants also completed a 30-min component known as the Cognitive Demand Battery (CDB), which comprises the prolonged repetition of a series of demanding tasks that assess working memory, executive function and attention. The objective of this battery is to assess the impact of treatment on speed/accuracy and mental fatigue during continuous performance of cognitively demanding tasks. The CDB has also been shown to be sensitive to modulation by a wide range of nutritional interventions [43,44,45,46]. The individual tasks making up the cognitive assessment (including the CDB) are shown in Figure 2 and described in more detail in the online Supplementary Materials (Section I).

Figure 2 also shows the contribution of individual tasks to the principal performance measures, which were derived by averaging the data (either msec for speed, or % correct/maximum score for accuracy) from individual tasks into the following global performance outcomes: ‘Speed of Performance’ and ‘Accuracy of Performance’; and the following cognitive domain factor scores ‘Speed of Attention’, ‘Accuracy of Attention’, ‘Speed of Memory’, ‘Working Memory’, and ‘Episodic Memory’. The derivation of the global scores and cognitive factors are described in more detail in the online Supplementary Materials (Section II). These global measures and cognitive domain factors have been shown to be sensitive to nutritional manipulations previously [40,41,42].

#### 2.4.2. Mood and Psychological State

Before each cognitive assessment, participants completed the Profile of Mood States (POMS-2) Adult Short Form [47]. As part of the COMPASS battery, and before the cognitive tasks, participants completed the Visual Analogue Mood Scales (VAMS), a set of 18 visual analogue scales anchored by pairs of antonymic mood/state adjectives (e.g., Alert–Inattentive; Lethargic–Energetic). Participants rated where they would position themselves between the adjectives anchoring each line according to how they felt at that moment. The individual item scores were combined to give an average (% along the line) score on three factors that had previously been derived by factor analysis: ‘Alertness’, ‘Tranquillity’ and ‘Stress’. After the cognitive tasks participants also completed a further four stress visual analogue scales (S-VAS) that required them to rate their current psychological state between ‘not at all’ and ‘extremely’ with regard to their levels of stress, anxiety, calmness and relaxation. These were combined into two scores ‘stress/anxiety’ and ‘calm/relaxed’ with a higher score (average % along the line) representing more of the descriptor.

### 2.5. Procedure

Participants were required to attend the Brain, Performance and Nutrition Research Centre (Northumbria University) for three visits. The first visit comprised a screening and training session where, once written informed consent had been obtained, participants were screened according to the inclusion/exclusion criteria. Eligible participants then provided lifestyle and demographic data and their height, weight, waist to hip ratio and blood pressure were measured. They completed a short training session in which they practiced the cognitive tasks. Practice took the form of three repetitions of shortened versions of the COMPASS cognitive tasks, followed by the completion of the full-length, 60-min battery twice. During and at the end of the practice session, participants’ performance was checked against standard minimum performance criteria and additional guidance was provided as necessary. At the end of this visit, participants were briefed as to what to expect on testing visits and were provided with pre-testing instructions.

**Figure 3:**
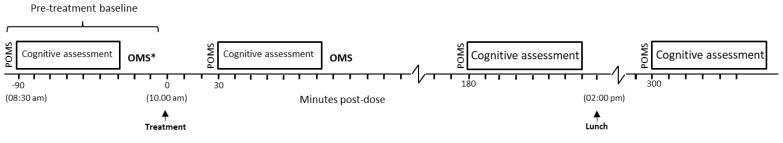
The timelines of the testing day for individual participants, showing the core cognitive assessment schedule. Profile of Mood States (POMS), 5 min Observed Multi-Tasking Stressor (*OMS) (methodology and results to be reported elsewhere).

Within four weeks of the screening visit, participants returned to the laboratory for their first testing visit at an agreed time in the morning that remained consistent across all testing visits. A maximum of 5 participants were tested on any day, and all participants were visually isolated in individual testing booths. Participants arrived at the laboratory having refrained from alcohol for 24 h, caffeine overnight and having consumed a simple breakfast of cereal and/or toast at home no later than an hour before arrival. Once participants arrived at the lab, they were not permitted to eat any food (aside from food items provided by the study staff) or drink (except for water) or chew gum. Continued compliance with the inclusion/exclusion criteria was assessed. This was followed by completion of the POMS and a 60-min computerised cognitive and mood assessment (COMPASS—including the 30-min Cognitive Demand Battery (CDB), Visual analogue mood scales (VAMS) and stress visual analogue scales (S-VAS)—see Figure 2.). Cognitive tasks were completed with the participants visually isolated from each other. After the first cognitive/mood assessment, participants consumed their treatment for the day and completed cognitive/mood assessments, identical to the above, commencing at 30 min, 3 h and 5 h post-dose. An additional, brief, 5-min assessment investigating the participants’ response to a laboratory stressor, plus pre/post-dose blood sampling for half of the participants (for quantification of neurotrophins and catecholamines), took place after the pre-treatment and 30-min post-dose cognitive/mood assessments (For methodology see [48]), the results of this theoretically distinct investigation are to be reported elsewhere). All participants were scheduled to return to the laboratory 7 days later, with a maximum allowable leeway of an additional 7 days should exceptional circumstances arise in the meantime. This second testing day was identical to the previous day, with the exception that participants consumed a different treatment on each of the two days. The timelines of the testing day are presented in Figure 3.

Participants were provided with a standardised lunch (comprising a cheese sandwich on white bread, crisps and a custard pot) between the 180 and 300 min post-dose assessments and were given the option of a snack (hot decaffeinated tea or coffee and digestive biscuits) after completion of the stressor following the 30-min post-dose assessment. No alternative drinks, snacks or lunches were permitted.

### 2.6. Analysis

The study statistical analysis plan was formulated before the completion of data collection. Given the exploratory nature of the study, and the lack of any relevant human data, a small sub-set of primary outcomes was not pre-defined. Given the study intervention and objectives, a per protocol analysis was deemed the most appropriate.

All outcomes were analysed using SPSS (version 24.0, IBM corp., Armonk, NY, USA). During blind data review a number of participants’ individual task datasets were removed due to technical or performance issues (for details of the issues and number of datasets involved see supplementary online materials). Prior to the primary analysis of the effects of treatment, pre-dose baseline differences between treatment were investigated by one-way (treatment group [placebo v mango leaf extract]) paired t tests, or in the case of the Cognitive Demand Battery (CDB) two-way (treatment x repetition) ANOVA. There were no significant differences between treatment groups at baseline.

For all cognitive and mood measures, the primary analysis of post-dose data was by Linear Mixed Models (LMM) using the MIXED procedure in SPSS (version 22.0, IBM corp.) with pre-dose baseline data for each outcome included as a covariate. For all LMM analyses, the ‘compound symmetry’ covariance structure provided the best fit, with the exception of ‘mental fatigue’ from the CDB for which an autoregressive covariance structure (AR1) was more appropriate.

For the cognitive outcomes derived from the COMPASS battery and the mood outcomes, terms were fitted for treatment (placebo/mango leaf extract) and assessment (30 min, 3 h, 5 h) and their interaction. For the CDB measures an additional ‘repetition’ term was added along with the appropriate interactions. Given that the treatment orders were balanced across the sample, or exactly or nearly balanced with regard to the participants contributing to each outcome (and given that treatment carry-over effects were highly unlikely), treatment order was not included as a factor in the analysis.

In order to establish the time course of any effects, pre-defined planned comparisons were conducted between treatments at each assessment time point (30 min, 3 h, 5 h) with a Bonferroni adjustment for the number of comparisons undertaken per outcome (i.e., 3). Only those planned comparisons conducted on data from outcomes that evinced a significant treatment related main or interaction effect are reported below.

## 3. Results

### 3.1. Cognitive Task Global and Factor Outcomes

**Figure 4:**
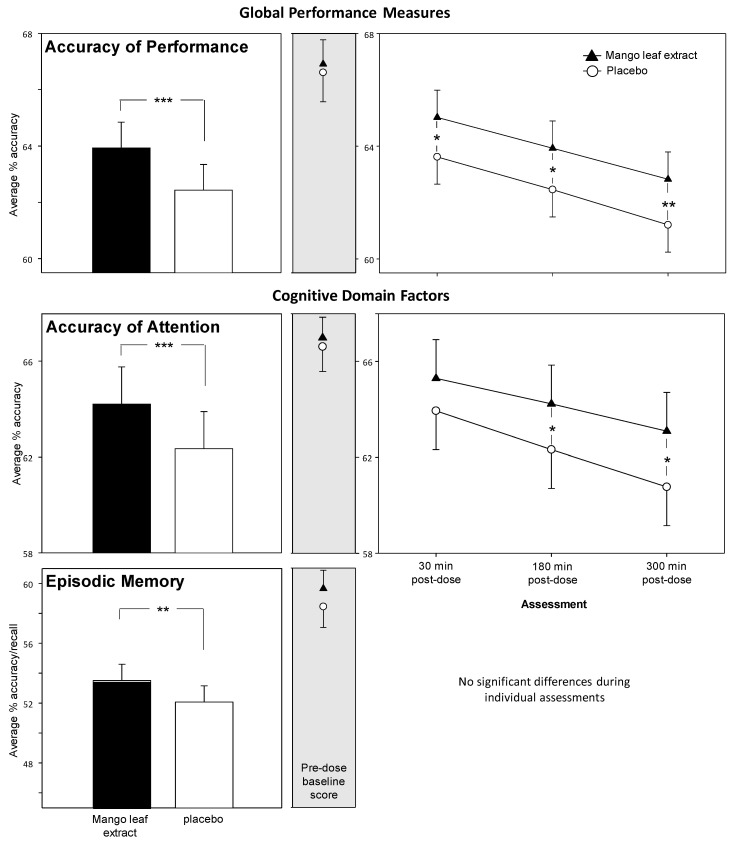
The effects of mango leaf extract on the global outcome measures and factor scores derived from the Computerised Mental Performance Assessment System (COMPASS) cognitive tasks. Left-hand panels show the main effect of treatment averaged across assessments; middle panels show the pre-dose baseline scores; right-hand panels show time course data from each post-dose assessment for those measures that saw significant effects on the planned comparisons (Bonferroni). The global Accuracy of Performance measure represents averaged data from the eleven tasks from the battery that return % accuracy/maximum score data: Accuracy of Attention represents averaged % accuracy data from the five attention tasks; and Episodic Memory represents averaged % accuracy/recall across the four long-term memory tasks. *, *p* < 0.05; **, *p* < 0.01, ***, *p* < 0.001 versus placebo. Number of participants contributing to the measure: Accuracy of Performance, *n* = 68, Episodic Memory, *n* = 70, Accuracy of Attention, *n* = 64.

The global outcomes and cognitive factors derived from the COMPASS battery showed that mango leaf extract resulted in significantly improved accuracy of performance across tasks and throughout the testing day (i.e., at 30 min, 3 h and 5 h post-dose). See Figure 4 below. There was a main effect of treatment on the global Accuracy of Performance measure (representing data from the eleven tasks that return % accuracy/maximum score data) (F (1, 335) = 22.8, *p* < 0.001). Reference to the planned comparisons at each assessment showed that this effect was evident throughout the post-dose testing period (30 min *p* = 0.03, 3 h *p* = 0.02, 5 h *p* = 0.009). There were also significant main effects in terms of improved accuracy following mango leaf extract on the Accuracy of Attention factor (F (1, 315) = 16.697, *p* < 0.001) and the Episodic Memory factor (F (1, 345) = 6.94, *p* = 0.009). With regard to the time course of these effects, whilst the Bonferroni adjusted comparisons of Episodic Memory scores did not reach significance during the individual assessments, Accuracy of Attention was improved at both the 3 h (*p* = 0.048) and 5 h (*p* = 0.01) post-dose assessments. Data (plus F score and *p*) for the cognitive outcomes derived from the COMPASS battery are presented in the online Supplementary Materials (Table S1.).

### 3.2. Cognitive Demand Battery (CDB)

**Figure 5:**
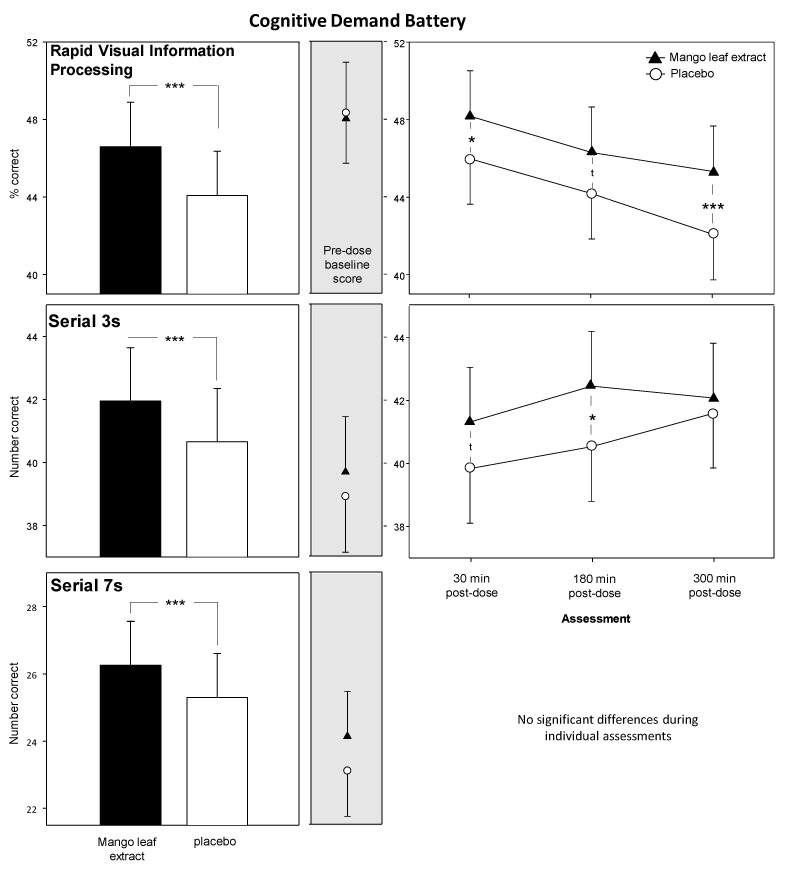
The effects of mango leaf extract on the Cognitive Demand Battery outcomes. Each task was repeated three times per assessment (total Cognitive Demand Battery (CDB) completion time, 30 min per assessment). Left-hand panels show the main effect of treatment averaged across assessments/repetitions; middle panels show the pre-dose baseline scores averaged across the three repetitions; right-hand panels show time course data from each post-dose assessment (averaged across the three repetitions per assessment) for those measures that saw significant effects on the planned comparisons (Bonferroni) of mango leaf extract versus placebo. t, *p* < 0.1; *, *p* < 0.05; ***; *p* < 0.001 in comparison to placebo. Number of participants contributing to the measure: RVIP, *n* = 64, Serial 3s/7s, *n* = 69.

In keeping with the improved accuracy seen across the COMPASS task factors, performance in all three CDB tasks was improved across the testing day following mango leaf extract. See Figure 5. The Rapid Visual Information Processing task (RVIP) was improved across assessments in terms of % of targets accurately detected (F (1, 1071) = 23.186, *p* < 0.001) with planned comparisons showing that these effects were apparent at the 30 min (*p* = 0.047) and 5 h (*p* = 0.001) assessments, with a trend towards the same effect at 3 h post-dose (*p* = 0.059). Performance was also improved on both the Serial 3s task (F (1, 1156) = 10.9, *p* < 0.001) and Serial 7s task (F (1, 1156) = 9.642, *p* = 0.002) in terms of number of correct subtractions across the testing day. Comparisons at each assessment showed that while the differences between groups did not reach significance during any individual assessment for the Serial 7s task, Serial 3s performance was enhanced at the 3 h assessment (*p* = 0.014), with a trend towards the same at 30 min post-dose (*p* = 0.088). There was no effect on ratings of mental fatigue during completion of the battery. Data (plus F score and *p*) for the CDB outcomes are presented in the online Supplementary Materials (Table S2.).

### 3.3. Mood and Psychological State

**Figure 6:**
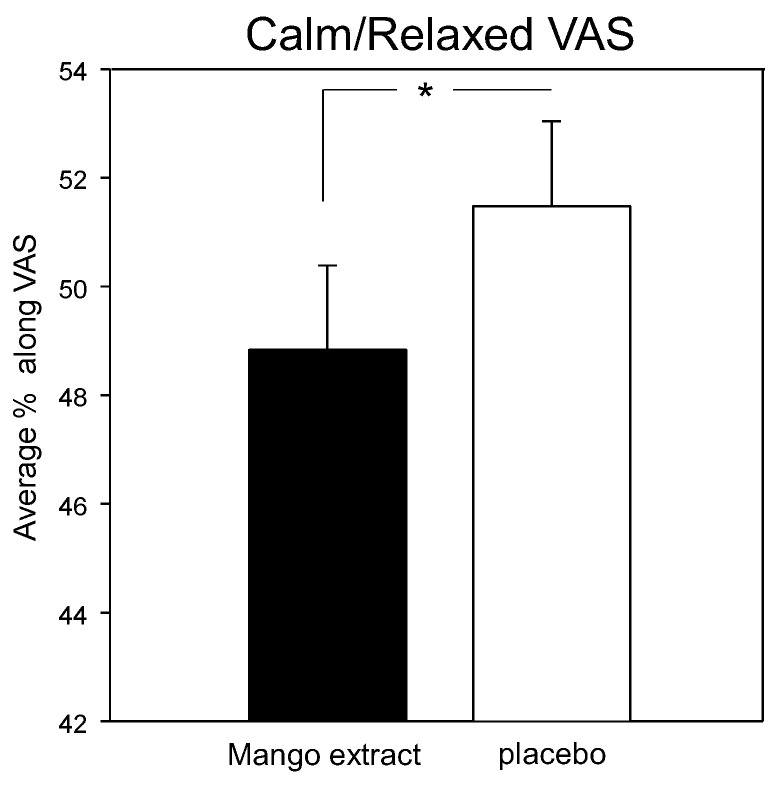
The effects of mango leaf extract on the calm/relaxed stress visual analogue scales (S-VAS) measure. There were no significant differences on the planned comparisons of data from each assessment. *, *p* < 0.05 in comparison to placebo.

There were no effects of treatment on any mood parameter (VAMS, S-VAS, POMS), with the exception of reduced calm/relaxed ratings on the S-VAS following mango leaf extract across testing assessments (F (1, 345) = 5.44, *p* = 0.02). See Figure 6. There were no significant differences on the comparisons made at each assessment for this outcome. Data from the POMS, VAMS and S-VAS data are presented in the online Supplementary Materials (Table S3.).

## 4. Discussion

In the current study a single dose of mango leaf extract (Zynamite^®^) lead to significant, broad improvements in performance across a battery of cognitive tasks throughout the 6 h following consumption. There were no interpretable benefits found for any measure of mood/psychological state.

Cognitive improvements were seen on the global Accuracy of Performance measure, which comprised averaged % accuracy or % maximum score data from 11 computerised tasks. It was also seen more specifically in the cognitive sub-factors ‘Accuracy of Attention’, representing the overall % accuracy whilst performing the five attention tasks (excludes simple reaction time) within the battery and ‘Episodic Memory’, which represents the % recall or accuracy of the four long-term memory tasks. Performance benefits were also seen across all three of the tasks that make up the 30-min Cognitive Demand Battery, with improved RVIP accuracy and increased numbers of correct subtractions generated by participants on both the Serial 3s and Serial 7s tasks. These cognitive effects, taken as a whole, were evident as main effects across the post-dose testing day, which comprised 60 min assessments starting at 30 min, 3 h and 5 h post-dose, without any clear pattern of augmentation or attenuation over time. There were no benefits seen in terms of increased speed of task performance on the timed tasks, or indeed on the mood and psychological state measures.

Clearly, one question raised by these results is whether the effects seen here represent a truly global improvement in accuracy across cognitive domains, or whether they simply reflect the consequences of improved attention. Certainly, attention and episodic memory are inter-related, with enhanced attention leading to improved encoding and retrieval of information. It has been suggested that episodic memory processes are themselves, to an extent, ‘acts of attention’ [49]. As the attention and episodic memory tasks comprised the majority of the tasks that contributed to the global accuracy measure, it is possible that the improvements to the latter are simply a reflection of broad improvements to attention. However, the improvements in Serial 3s and Serial 7s subtraction task performance would be more difficult to accommodate solely within an attention framework. Whilst both subtraction tasks have attentional components, they draw more heavily on both working memory and executive function, particularly the more difficult Serial 7s, which requires greater executive resources in order to carry out the more complex manipulation of numbers [24]. Enhanced performance on these tasks, alongside improved accuracy across the tasks, therefore, seems to confirm that the benefits of mango leaf extract were seen broadly across cognitive domains.

The results also suggest that the modulation of cerebro-electrical activity (measured using EEG) seen in healthy adults following a single dose of Zynamite mango leaf extract [39] is most likely indicative of a benefit to brain function. The cognitive benefits seen here are broadly in line with previous demonstrations of improved cognitive function following both acute [24,25,26] and chronic administration [27,28] of polyphenol rich extracts. Several polyphenol studies also employed the Cognitive Demand Battery used here (but at a single post-dose time point), with demonstrations of improved performance across all three tasks following cocoa-flavanols [24], improved Serial 3s performance following fruit flavanols [50], but no benefits following resveratrol [51]. Of note, the global performance measures derived from the cognitive tasks utilised here have proved sensitive to the acute and chronic administration of a Nepalese pepper extract [42] and acute administration of a green oat extract to middle-aged adults [41]. However, both of these interventions contain other potentially bioactive phytochemicals alongside polyphenols, and in both cases global speed of performance was enhanced, rather than the improved global accuracy seen in the current study.

Previous research has demonstrated similarities in EEG cerebro-electrical response following both mango leaf extract and caffeine in rodents [38], but somewhat different responses to these two individual treatments in humans [39]. The cognitive effects of caffeine comprise modest but consistent improvements that are restricted to the performance of tasks measuring attention, with no reliable effect on other cognitive domains including long-term (episodic) memory [52,53,54,55]. Similarly, the duration of the effects seen following mango leaf extract do not follow the time course of caffeine’s effects, which would become apparent by 30 min post-dose and would be expected to attenuate by 6 h post-dose. It is therefore notable that the pattern of cognitive benefits seen in the present study following the mango leaf extract are broader and longer lasting than those that would be expected after caffeine.

In terms of mechanism of action, a recent study investigating receptor binding and brain relevant enzyme inhibition found that mangiferin only significantly inhibited catechol-O-methyl transferase (COMT), the enzyme responsible for the degradation of catecholamine neurotransmitters [39]. Several other polyphenols that also feature a catechol moiety, including flavanols and oleacein, have also been shown to inhibit COMT [56,57]. COMT’s catabolic pathway is most prevalent in brain tissue with low concentrations of catecholamine reuptake transporters, and therefore COMT inhibition predominantly affects dopaminergic function in the prefrontal cortex and hippocampus [58], potentially leading to improved working memory, selective attention, and executive function [59]. Clearly, the benefits seen in the current study correspond with these cognitive domains. However, whilst there is some evidence that COMT inhibitors may modulate these aspects of cognitive function, the overall pattern is for their effects to be bidirectionally moderated by COMT genotype (val158met polymorphism) [59,60,61]. COMT inhibition per se is therefore unlikely to be the primary mechanism underpinning the straightforward cognitive benefits seen here across a sample of mixed COMT genotypes.

Other potential ‘direct’ brain-relevant mechanisms of action previously established for mangiferin include acetylcholinesterase (AChE) inhibition [30,62] or other potential cholinergic mechanisms of action [63]. Increased acetylcholine activity would be expected to have a beneficial, inter-related effect on both focussed attention and memory consolidation/retrieval [64] and, therefore, could encompass many of the effects seen in the current study. However, it is equally likely that the effects seen here may be related to ‘indirect’ interactions within mammalian cellular signal transduction pathways, a property that mangiferin shares with other polyphenols [8,11,12,13,14]. These interactions potentially drive downstream modulation of neuroinflammation, neurotransmission, neurotrophin receptor and signalling pathway interactions, and increased synthesis of neurotrophins and vasodilatory molecules, leading to increased angiogenesis/neurogenesis and local cerebral blood flow [15,16,17,18,19]. These indirect cellular interactions may underlie the consistent demonstrations in humans of increased cerebral blood-flow [51,65,66,67,68,69] and peripherally measured brain-derived neurotrophic factor [26] seen following diverse polyphenols. Again, potentially diffuse beneficial effects within the brain could be conceived as potentially leading to broad benefits to cognitive function across domains, as seen here.

Clearly, a strength of the current study is that it represents the first concerted investigation of the effects of mangiferin, or indeed any xanthone glycoside, on human cognitive function. Conversely, this was, by its nature, an exploratory study, and the absence of pre-defined primary outcomes, due to a lack of previous data to guide their formulation, could be considered a limitation. Certainly the absence of primary endpoints allows a greater freedom for the interpretation of the results than will be enjoyed in future research, and it is hoped that the results of the current study will be useful in terms of directing the research questions and outcomes addressed by more studies involving this compound. 

It should also be acknowledged that the results herein relate to a molecule, or group of molecules (xanthones) that are unlikely to be encountered in meaningful quantities in the typical diet, and therefore the results can only realistically be extrapolated to supplementation with mangiferin-rich extracts. Whilst the results tell us little about the benefits of polyphenols consumed as part of the everyday diet it might be noteworthy that the dose of 300 mg employed here contained an amount of polyphenols that is achievable through the consumption of polyphenol rich foods.

In conclusion, a single dose of mango leaf extract (Zynamite^®^) with high levels of the polyphenol mangiferin, lead to broad improvements in cognitive function that were seen across assessments spanning from 30 min to 6 h post-dose. These benefits were seen most strikingly in terms of participants’ improved attention and long-term memory task performance and in their extended performance of cognitively demanding tasks, including those requiring executive function resources.

## Supplementary Materials

The following are available online at https://www.mdpi.com/2072-6643/12/8/2194/s1. Section I: Individual COMPASS and CDB cognitive task descriptions. Section II: Derivation of the global outcome measures and cognitive domain factors, including notes on lost data. Section III: Table S1. (data from global measures and cognitive factors), Table S2. (data from Cognitive Demand Battery), Table S3. (data from the mood measures).
