# Supplementary material for: Acute Effects of a Polyphenol-Rich Leaf Extract of Mangifera indica L. (Zynamite) on Cognitive Function in Healthy Adults: A Double-Blind, Placebo-Controlled Crossover Study
Source: Nutrients. 2020 Jul 23;12(8):2194. doi: 10.3390/nu12082194 (PMC7468873; doi:10.3390/nu12082194)
Supplement: Supplementary file 1 [file nutrients-12-02194-s001.pdf]

Acute effects of a polyphenol-rich leaf extract of *Mangifera indica* L. (Zynamite) on cognitive function in healthy adults: a double-blind, placebo-controlled, cross-over study.

<u>**Online Supplementary Materials**</u>

SECTION I – INDIVIDUAL COMPASS COGNITIVE TASK DESCRIPTION

Cognitive function was assessed using the Computerised Mental Performance Assessment System (COMPASS – Northumbria University). This testing system delivers a bespoke collection of tasks, with fully randomised parallel versions of each task delivered at each assessment for each individual. It has previously been shown to be sensitive to a wide range of nutritional interventions [1-4] . The selection of tasks employed here comprised a number of standard and 'classic' tasks that assess aspects of memory (working, episodic, spatial), attention and executive function.

'Episodic' long-term Memory Tasks:

Picture presentation: Fifteen colour photographic images of objects will be presented sequentially on screen for the participant to remember at the rate of 1 every 3 s, with a stimulus duration of one second.

Word Presentation: A unique set of fifteen words will be presented. Words will be selected at random from a large bank of words (MRC Psycholinguistic Database) matched for word length, frequency, familiarity and concreteness. Stimulus duration will be one second, as will be the inter-stimulus duration.

Immediate and Delayed Word Recall: Participants write down as many of the 15 words that they were presented during the stimulus presentation period immediately after this period and also during the delayed recall/recognition period. This task is scored for accuracy only.

Delayed Picture Recognition: A series of pictures are displayed on the screen, one at a time. The number of pictures, the rate at which they are displayed and the interstimulus interval can be modified. All target pictures shown during Picture Presentation plus an equal number of decoys will be displayed on the screen one at a time. For each stimulus participants select 'Yes' or 'No' to indicate if they have seen the picture before or not. The task outcomes include accuracy and reaction time.

Attention:

Digit Vigilance Task: A target digit is randomly selected and constantly displayed to the right of the computer screen. A series of digits is then presented in the centre of the screen at the rate of 80 per minute and the participant is required to press the YES button as quickly as possible every time the digit in the series matches the target digit. The task lasts 2 min and there are 30 stimulus–target matches. Task outcomes are accuracy, mean reaction time and number of false alarms.

Choice Reaction Time: an arrow appears on the screen pointing to the left or to the right. Participants respond with a left or right key press corresponding to the direction of the arrow. There is a randomly varying inter-stimulus interval of between 1 and 3 seconds for a total of fifty stimuli. The task outcomes are accuracy and mean reaction time for correct responses.

Simple Reaction Time: Participants respond with a single key press every time a upwards pointing arrow appears on the screen. There was a randomly varying inter-stimulus interval of between 1 and 3 seconds for a total of fifty stimuli. Task outcomes are accuracy and mean reaction time.

Working Memory:

'Sternberg' Numeric Working Memory task: A series of five single digits are displayed on the screen, one at a time. Participants are required to try to memorise these numbers as they appear. Once the series is complete, 30 single digits will be displayed one at a time and participants will respond to indicate if each number was presented in the previous list or not. The task will be repeated three times with different digits. The task outcomes include accuracy and reaction time.

'Corsi Blocks': Spatial Working Memory Task. Nine blue squares on a black background are displayed on the screen. Some of the blue squares change to red and back to blue again in a sequence. Participants are required to remember this sequence. The task is repeated five times at each level of difficulty with the sequence span increasing from 4 upwards, until the participant can no longer correctly recall the sequences. The task outcome is 'span score' and this is calculated as the average of the last 3 correctly completed trials. For example, if the participant correctly responds to all five Level 4 trials and only one Level 5 trial, their span score would be 4.3 [(4 + 4 + 5)/3].

Executive Function

Peg and Ball: Two configurations will be shown on the screen. In each there will be three coloured balls (blue, green, red) on one of 3 pegs. The configuration at the top of the screen is the goal configuration and participants must arrange the balls on the starting configuration (shown in the centre of the screen) to match the position of balls in the goal configuration. They must do this in the least number of moves possible. Task outcomes include average thinking time, completion time and errors.

COGNITIVE DEMAND BATTERY (CDB)

The battery of tasks also included extended performance of the CDB. The objective of this battery is to assess the impact of treatment on speed/accuracy and mental fatigue during continuous performance of cognitively demanding tasks.

Participants complete the 10 minute battery of tasks three times in immediate succession (i.e. for a continuous period of 30 minutes). Application of this battery has been shown to reliably increase self-ratings of 'mental fatigue' and to be sensitive to a number of herbal and natural interventions [5-10].

The 10-minute battery comprises:

Serial 3s subtraction task (2 mins): Computerised versions of the working memory/executive function serial subtraction tasks are implemented using tests of 2-minute duration. Participants are required to count backwards in threes from a given number as quickly and as accurately as possible using the

linear keyboard number keys to enter each response. A random starting number between 800 and 999 is presented on the computer screen, which is cleared by the entry of the first response. The task is scored for number of correct responses and number of errors. In the case of incorrect responses subsequent responses are scored as positive if they were scored as correct in relation to the new number.

Serial 7s subtraction task (2 mins): This is identical to the serial threes task with the exception that it involves the serial subtraction of sevens.

Rapid Visual Information Processing task (RVIP – 5 mins): In this classic test of focussed attention, the participant is required to monitor a continuous series of digits for targets of three consecutive odd or three consecutive even digits. The digits are presented at the rate of 100 per minute and the participant responds to the detection of a target string by pressing the response button as quickly as possible. The task is continuous and lasts for 5 minutes, with 8 correct target strings being presented in each minute. The task is scored for percentage of target strings correctly detected, average reaction time for correct detections, and number of false alarms.

'*Mental fatigue' visual analogue scale*; Participants rate their current subjective 'mental fatigue' state by making a mark on a 100 mm line with the end points labelled "not at all" (left hand end) and "very much so" (right hand end).

SECTION II: DERIVATION OF GLOBAL OUTCOMES AND COGNITIVE FACTORS

The outcomes from the COMPASS battery can be collapsed into the global performance measures (global Speed of Performance and Accuracy of Performance) and cognitive 'factors' (Speed of Attention, Accuracy of Attention, Speed of Memory, Working Memory, Episodic Memory) which can be useful to establish if the treatment has a global effect or an effect restricted to given cognitive domains that might escape significance on the component tasks. The task order and the outcomes contributing to the factor scores are shown in Figure 1. (main paper). Similar factors have been shown to be sensitive to diverse nutritional manipulations previously [1,3,11]. The derivation of the global outcomes and factors is described below.

Global measures

'Speed of Performance': this global measure represents the average speed of performance (msec) of all of the tasks within the battery that collect reaction time data to individual stimuli : Choice Reaction Time, Simple Reaction Time, Digit Vigilance Task, Rapid Visual Information Processing (x 3), Numeric Working Memory, Delayed Picture Recognition, Delayed Word Recognition.

'Accuracy of Performance': this global measure represents the average % accuracy of all of the tasks for which this data is calculable: Choice Reaction Time (% correct responses), Digit Vigilance Task (% targets correctly identified), Rapid Visual Information Processing x 3 (% targets correctly identified), Numeric Working Memory (% correct responses), Corsi Blocks (% of the maximum score), Immediate Word Recall (% of the maximum score), Delayed Word Recall (% of the maximum score), Delayed Picture Recognition (% correct responses), Delayed Word Recognition (% correct responses).

Cognitive Domain Factors

'Speed of Attention': this measure represents the average speed (msecs) of performing the attention tasks within the battery that collect reaction time data to individual stimuli. Choice Reaction Time, Simple Reaction Time, Digit Vigilance Task, Rapid Visual Information Processing (x 3).

'Accuracy of Attention': this measure represents the average % accuracy of performing the attention tasks within the battery: Choice Reaction Time (% correct responses), Digit Vigilance Task (% targets correctly identified), Rapid Visual Information Processing x 3 (% targets correctly identified).

'Speed of Memory': this measure represents the average speed (msec) of performing the working and episodic memory tasks that collect reaction time data to individual stimuli: Numeric Working Memory, Delayed Picture Recognition, Delayed Word Recognition.

'Working Memory': this measure represents the average % accuracy/maximum score of performing the working memory tasks within the battery: Numeric Working Memory (% correct responses), Corsi Blocks (% of the maximum score)

'Episodic Memory': this measure represents the average % accuracy/maximum score of performing the long-term memory tasks within the battery: Immediate Word Recall (% of the maximum score), Delayed Word Recall (% of the maximum score), Delayed Picture Recognition (% correct responses), Delayed Word Recognition (% correct responses).

In the case of loss of data for a participant on individual tasks (see next section), data from all repetitions of that task was removed and the average global outcome and cognitive factor scores were calculated by averaging the scores from the remaining tasks. The maximum allowable data loss was 30% of task outcomes contributing to the measure.

SECTION III: TABLES

			Pre-dose Baseline		Post-dose data (averaged)		Individual post-dose assessment data: 30 min		3 h		5 h				
		n	mean	sem	mean	sem	mean	sem	mean	sem	mean	sem		Fs	p
**Global performance measures**															
**Accuracy of Performance**	300 mg	68	66.99	0.86	63.93	0.92	65.03	0.97	63.93	0.97	62.83	0.97	T	22.83	<0.001
Average % correct/max score (11 tasks)	Placebo		66.62	1.04	62.43	0.92	63.62	0.97	62.46	0.97	61.21	0.97	TxA	0.04	-
**Speed of Performance**	300 mg	68	616.8	9.57	607.6	7.95	610.9	8.54	610.7	8.54	601.3	8.54	T	0.94	-
Average speed in msec (10 tasks)	Placebo		605.9	8.34	604.6	7.95	609.7	8.54	605.4	8.54	598.7	8.54	TxA	0.15	-
**Cognitive domain factors**															
**Accuracy of Attention**	300 mg	64	65.87	1.36	64.21	1.55	65.30	1.62	64.23	1.62	63.09	1.62	T	16.70	<0.001
Average % correct (5 tasks)	Placebo		66.05	1.61	62.34	1.55	63.94	1.62	62.32	1.62	60.77	1.62	TxA	0.38	-
**Speed of Attention**	300 mg	64	483.5	3.88	485.2	4.19	486.0	4.54	485.1	4.54	484.5	4.54	T	0.16	-
Average speed in msec (6 tasks)	Placebo		481.4	4.22	484.5	4.19	485.2	4.54	484.9	4.54	483.4	4.54	TxA	0.02	-
**Episodic Memory**	300 mg	70	59.64	1.22	53.51	1.09	54.77	1.22	53.40	1.22	52.36	1.22	T	6.94	0.01
Average % recall (4 tasks)	Placebo		58.43	1.40	52.07	1.09	53.32	1.22	51.97	1.22	50.92	1.22	TxA	0.00	-
**Working Memory**	300 mg	61	82.44	0.70	82.00	0.62	82.41	0.74	82.50	0.74	81.10	0.74	T	0.69	-
Average % correct (2 tasks)	Placebo		82.34	0.61	81.66	0.62	81.34	0.74	82.18	0.74	81.47	0.74	TxA	1.02	-
**Speed of Memory**	300 mg	68	891.0	22.8	859.8	17.4	869.2	19.5	867.6	19.5	842.8	19.5	T	0.64	-
Average speed in msec (3 tasks)	Placebo		861.9	18.9	852.9	17.4	866.7	19.5	853.3	19.5	838.6	19.5	TxA	0.18	-

Table S1. The effects of mango leaf extract on the global outcome measures and cognitive domain factor scores derived from the COMPASS cognitive tasks. Baseline data are raw scores (+SEM) and post-dose data are estimated means (+SEM) from the LMM analysis. The right-hand columns contain the F scores and probabilities from the LMM analysis. T = main effect of treatment, TxA = treatment x assessment interaction

Data loss: at blind data review, and prior to the calculation of global performance and factor scores, the individual task data for 7 participants were removed from one or more of 5 tasks due to data inconsistencies across more than one repetition of the task. The inconsistencies took the form of: a lack of any RVIP data, suggesting either an issue with the button box (pressing wrong key, box malfunction) or that the participant was not performing the task; very high levels of false alarms during the RVIP task suggesting repetitive random responses; chance or significantly below chance performance on a task (Numeric working memory, Word recognition, Corsi blocks) suggesting random performance or incorrect button responses. The total number of lost datasets were: RVIP x 6, Corsi blocks x 2, NWM x 7, Word recognition x 5, CRT. The global outcome and factor scores were recalculated (averaged) using the remaining individual task outcomes, provided the data loss did not exceed 30% of the task outcomes contributing to the measure. The number of datasets contributing to each outcome is shown above.

	Repetition		N	Pre-dose Baseline mean	sem	Individual post-dose assessment 30 min mean	sem	3 hr mean	sem	5 hr mean	sem		Fs	p
**Mental Fatigue** (% along VAS)	Rep 1	300 mg	69	53.35	2.07	62.70	1.99	65.48	1.99	69.42	1.99	T	1.15	-
	Rep 2			60.14	2.06	68.45	1.99	72.30	1.99	76.87	1.99	TxA	0.48	-
	Rep 3			66.83	2.40	71.54	1.99	74.49	1.99	80.03	1.99	TxR	0.05	-
	Rep 1	Placebo	69	52.00	2.15	59.59	1.99	62.97	1.99	69.78	1.99	TxRxA	0.53	-
	Rep 2			60.93	2.03	67.44	1.99	69.03	1.99	75.99	1.99			
	Rep 3			66.06	2.23	70.33	1.99	71.57	1.99	77.97	1.99			
**RVIP** (% correct)	Rep 1	300 mg	64	51.25	2.30	51.33	2.52	48.09	2.52	48.13	2.52	T	23.19	<0.001
	Rep 2			46.48	2.32	47.46	2.52	46.29	2.52	43.59	2.52	TxA	0.49	-
	Rep 3			46.33	2.17	45.74	2.52	44.53	2.52	44.26	2.52	TxR	0.04	-
	Rep 1	Placebo	64	51.17	2.50	49.30	2.52	46.02	2.52	44.77	2.52	TxRxA	0.03	-
	Rep 2			48.91	2.72	45.00	2.52	43.71	2.52	40.47	2.52			
	Rep 3			44.92	2.60	43.63	2.52	42.81	2.52	40.98	2.52			
**RVIP** reaction time (msec)	Rep 1	300 mg	64	532.07	6.22	528.12	6.70	523.53	6.70	528.41	6.70	T	0.01	-
	Rep 2			540.69	6.88	536.78	6.70	536.68	6.70	529.43	6.70	TxA	0.12	-
	Rep 3			544.33	6.46	533.80	6.70	539.15	6.70	532.00	6.70	TxR	0.69	-
	Rep 1	Placebo	64	531.88	6.46	532.60	6.70	530.16	6.70	527.18	6.70	TxRxA	0.84	-
	Rep 2			540.57	6.53	532.64	6.70	530.93	6.70	533.51	6.70			
	Rep 3			541.99	6.52	537.38	6.70	535.44	6.70	529.74	6.70			
**Serial 3s** Number correct	Rep 1	300 mg	69	39.48	1.78	40.87	1.87	42.75	1.87	42.94	1.87	T	10.99	0.001
	Rep 2			40.36	1.85	41.00	1.87	41.64	1.87	41.97	1.87	TxA	1.18	-
	Rep 3			39.17	1.64	42.09	1.87	42.99	1.87	41.33	1.87	TxR	0.66	-
	Rep 1	Placebo	69	39.87	1.85	40.57	1.87	40.41	1.87	42.96	1.87	TxRxA	0.56	-
	Rep 2			38.71	1.67	38.74	1.87	40.71	1.87	41.88	1.87			
	Rep 3			38.12	1.84	40.22	1.87	40.49	1.87	39.94	1.87			
**Serial 3s** Errors	Rep 1	300 mg	69	2.45	0.24	2.97	0.35	2.44	0.35	2.86	0.35	T	2.03	-
	Rep 2			2.72	0.37	2.93	0.35	2.36	0.35	2.48	0.35	TxA	1.52	-
	Rep 3			2.94	0.39	2.55	0.35	2.20	0.35	2.84	0.35	TxR	1.55	-
	Rep 1	Placebo	69	2.46	0.31	2.49	0.35	2.87	0.35	2.70	0.35	TxRxA	0.36	-
	Rep 2			2.43	0.37	2.75	0.35	2.55	0.35	2.88	0.35			
	Rep 3			2.71	0.35	2.91	0.35	3.10	0.35	3.17	0.35			
**Serial 7s** Number correct	Rep 1	300 mg	69	23.84	1.32	24.87	1.42	26.68	1.42	26.93	1.42	T	11.78	0.001
	Rep 2			24.16	1.31	26.19	1.42	26.00	1.42	26.77	1.42	TxA	0.00	-
	Rep 3			24.36	1.40	25.41	1.42	26.96	1.42	26.48	1.42	TxR	0.03	-
	Rep 1	Placebo	69	22.35	1.30	24.55	1.42	25.45	1.42	25.36	1.42	TxRxA	0.93	-
	Rep 2			23.07	1.38	24.81	1.42	25.81	1.42	25.68	1.42			
	Rep 3			23.75	1.39	24.22	1.42	25.46	1.42	26.33	1.42			
**Serial 7s** Errors	Rep 1	300 mg	69	2.45	0.28	2.68	0.32	2.73	0.32	2.88	0.32	T	1.35	-
	Rep 2			2.75	0.26	2.42	0.32	2.97	0.32	2.64	0.32	TxA	0.44	-
	Rep 3			2.90	0.37	2.55	0.32	2.65	0.32	2.86	0.32	TxR	0.67	-
	Rep 1	Placebo	69	2.59	0.28	2.51	0.32	2.80	0.32	2.86	0.32	TxRxA	0.44	-
	Rep 2			2.71	0.26	2.80	0.32	3.01	0.32	2.73	0.32			
	Rep 3			3.04	0.35	3.22	0.32	2.74	0.32	2.94	0.32			

Table S2. Data from the Cognitive Demand Battery. Baseline data are raw scores (+SEM) and post-dose data are estimated means (+SEM) from the LMM analysis. Each assessment comprised three repetitions of the tasks. The right hand columns contain the F scores and probabilities from the LMM analysis. T = main effect of treatment, TxA = treatment x assessment interaction, TxR = treatment x repetition interaction, TxRxA = treatment x repetition x assessment interaction.

Data loss: at blind data review one participant was removed from the Serial subtraction tasks due to a lack of any correct responses during multiple repetitions.

			Pre-dose Baseline		Post-dose Averaged data		Individual post-dose assessment 30 min		3 hr		5 hr				
		n	mean	sem	mean	sem	mean	sem	mean	sem	mean	sem		Fs	p
Visual Analogue Mood Scales (VAMS)															
Alertness	300 mg	69	59.90	1.58	55.02	1.49	57.04	1.69	55.10	1.69	52.93	1.69	T	1.69	-
	Placebo		59.75	1.80	56.07	1.49	57.10	1.69	56.48	1.69	54.61	1.69	TxA	0.39	-
Stress	300 mg	69	39.87	1.44	41.84	1.31	41.66	1.49	42.05	1.49	41.82	1.49	T	0.15	-
	Placebo		39.43	1.69	41.56	1.31	41.83	1.49	40.90	1.49	41.96	1.49	TxA	0.38	-
Tranquility	300 mg	69	65.39	1.37	62.83	1.29	63.29	1.44	62.82	1.44	62.39	1.44	T	1.59	-
	Placebo		66.36	1.48	63.64	1.29	63.94	1.44	63.50	1.44	63.47	1.44	TxA	0.05	-
Stress Visual Analogue Mood Scales (S-VAS)															
Stressed/anxious VAS	300 mg	70	43.65	1.78	46.77	1.66	47.06	2.04	46.13	2.04	47.11	2.04	T	1.06	-
	Placebo		42.61	2.08	45.55	1.66	45.14	2.04	44.61	2.04	46.89	2.04	TxA	0.19	-
Calm/relaxed VAS	300 mg	70	50.86	1.66	48.84	1.46	50.16	1.83	47.69	1.83	48.66	1.83	T	5.44	0.02
	Placebo		50.79	1.90	51.40	1.46	51.14	1.83	51.26	1.83	51.80	1.83	TxA	0.53	-
Profile of Mood States (POMS)															
Friendliness	300 mg	68	13.00	0.34	11.54	0.49	12.15	0.53	11.55	0.53	10.91	0.53	T	1.98	-
	Placebo	68	13.81	0.34	11.26	0.49	11.82	0.52	11.24	0.53	10.73	0.53	TxA	0.05	-
Vigour	300 mg	68	9.16	0.44	7.89	0.46	8.09	0.50	8.10	0.50	7.49	0.50	T	2.17	-
	Placebo	68	10.06	0.44	7.62	0.46	8.35	0.50	7.69	0.50	6.82	0.50	TxA	2.21	-
Tension	300 mg	68	3.00	0.33	2.42	0.31	2.69	0.35	2.15	0.35	2.41	0.35	T	2.41	-
	Placebo	68	3.53	0.34	2.17	0.31	2.74	0.35	1.91	0.35	1.85	0.35	TxA	1.22	-
Fatigue	300 mg	68	4.05	0.38	5.43	0.47	4.82	0.52	5.34	0.53	6.13	0.52	T	0.23	-
	Placebo	68	4.25	0.38	5.32	0.47	4.96	0.52	4.92	0.52	6.09	0.52	TxA	0.48	-
Depression	300 mg	68	1.43	0.28	1.16	0.25	1.21	0.27	1.10	0.27	1.18	0.27	T	0.03	-
	Placebo	68	1.50	0.29	1.19	0.25	1.36	0.27	0.99	0.27	1.20	0.27	TxA	0.38	-
Confusion	300 mg	68	2.00	0.29	2.13	0.32	2.02	0.35	1.99	0.35	2.37	0.35	T	0.02	-
	Placebo	68	2.78	0.31	2.15	0.32	2.18	0.35	1.95	0.35	2.31	0.35	TxA	0.27	-
Anger	300 mg	68	1.68	0.25	1.24	0.22	1.14	0.25	1.21	0.25	1.37	0.25	T	1.67	-
	Placebo	68	1.78	0.27	1.09	0.22	1.21	0.25	0.99	0.25	1.07	0.25	TxA	1.00	-
Total Mood Disturbance	300 mg	68	3.00	1.36	4.48	1.55	3.79	1.68	3.70	1.68	5.97	1.68	T	0.09	-
	Placebo	68	3.78	1.43	4.29	1.54	4.11	1.68	3.08	1.68	5.69	1.68	TxA	0.18	-

Table S3. Data from the mood measures. Baseline data are raw scores (+SEM) and post-dose data are estimated means (+SEM) from the LMM analysis. Post-dose data are presented both averaged across the three post-dose assessments (30 min, 3 h, 5 h) and by the individual assessments. The right-hand columns contain the F scores and probabilities for significant results from the LMM analysis. T = main effect of treatment, TxA = treatment x assessment interaction.

REFERENCES

1. Stonehouse, W.; Conlon, C.A.; Podd, J.; Hill, S.R.; Minihane, A.M.; Haskell, C.; Kennedy, D. Dha supplementation improved both memory and reaction time in healthy young adults: A randomized controlled trial. *American Journal of Clinical Nutrition* **2013**, *97*, 1134-1143.
2. Kennedy, D.O.; Wightman, E.L.; Forster, J.; Khan, J.; Haskell-Ramsay, C.F.; Jackson, P.A. Cognitive and mood effects of a nutrient enriched breakfast bar in healthy adults: A randomised, double-blind, placebo-controlled, parallel groups study. *Nutrients* **2017**, *9*, 1332.
3. Kennedy, D.O.; Jackson, P.A.; Forster, J.; Khan, J.; Grothe, T.; Perrinjaquet-Moccetti, T.; Haskell-Ramsay, C.F. Acute effects of a wild green-oat (avena sativa) extract on cognitive function in middle-aged adults: A double-blind, placebo-controlled, within-subjects trial. *Nutr Neurosci* **2017**, *20*, 135-151.
4. Kennedy, D.O.; Dodd, F.L.; Robertson, B.C.; Okello, E.J.; Reay, J.L.; Scholey, A.B.; Haskell, C.F. Monoterpenoid extract of sage (salvia lavandulaefolia) with cholinesterase inhibiting properties improves cognitive performance and mood in healthy adults. *Journal of Psychopharmacology* **2011**, *25*, 1088-1100.
5. Kennedy, D.O.; Haskell, C.F.; Robertson, B.; Reay, J.; Brewster-Maund, C.; Luedemann, J.; Maggini, S.; Ruf, M.; Zangara, A.; Scholey, A.B. Improved cognitive performance and mental fatigue following a multi-vitamin and mineral supplement with added guarana (paullinia cupana). *Appetite* **2008**, *50*, 506-513.
6. Kennedy, D.O.; Scholey, A.B. A glucose-caffeine 'energy drink' ameliorates subjective and performance deficits during prolonged cognitive demand. *Appetite* **2004**, *42*, 331-333.
7. Reay, J.L.; Kennedy, D.O.; Scholey, A.B. Single doses of panax ginseng (g115) reduce blood glucose levels and improve cognitive performance during sustained mental activity. *J Psychopharmacol* **2005**, *19*, 357-365.
8. Reay, J.L.; Kennedy, D.O.; Scholey, A.B. Effects of panax ginseng, consumed with and without glucose, on blood glucose levels and cognitive performance during sustained 'mentally demanding' tasks. *J Psychopharmacol* **2006**, *20*, 771-781.
9. Kennedy, D.; Okello, E.; Chazot, P.; Howes, M.-J.; Ohiomokhare, S.; Jackson, P.; Haskell-Ramsay, C.; Khan, J.; Forster, J.; Wightman, E. Volatile terpenes and brain function: Investigation of the cognitive and mood effects of mentha× piperita l. Essential oil with in vitro properties relevant to central nervous system function. *Nutrients* **2018**, *10*, 1029.
10. Scholey, A.B.; French, S.J.; Morris, P.J.; Kennedy, D.O.; Milne, A.L.; Haskell, C.F. Consumption of cocoa flavanols results in acute improvements in mood and cognitive performance during sustained mental effort. *J Psychopharmacol* **2010**, *24*, 1505-1514.
11. Kennedy, D.; Wightman, E.; Khan, J.; Grothe, T.; Jackson, P. The acute and chronic cognitive and cerebral blood-flow effects of nepalese pepper (zanthoxylum armatum dc.) extract—a randomized, double-blind, placebo-controlled study in healthy humans. *Nutrients* **2019**, *11*, 3022.
